# Variation of Signal Reflection on Electrodes of Silicon Mach-Zehnder Modulators: Influence of Nanoscale Variation and Mitigation Strategies

**DOI:** 10.3390/nano11020499

**Published:** 2021-02-16

**Authors:** Zhaobang Zeng, Ding Ding, Qianyi Gao, Nan Yang, Peiyan Zhao, Wei Jiang

**Affiliations:** 1 College of Engineering and Applied Sciences, Nanjing University, Nanjing 210093, China; mg1534001@smail.nju.edu.cn (Z.Z.); mg1834003@smail.nju.edu.cn (D.D.); dz1934003@smail.nju.edu.cn (Q.G.); mg1634018@smail.nju.edu.cn (N.Y.); mg1434006@smail.nju.edu.cn (P.Z.); 2 Key Laboratory of Intelligent Optical Sensing and Manipulation, Nanjing University, Ministry of Education, Nanjing 210093, China; 3 National Laboratory of Solid State Microstructures, Nanjing 210093, China

**Keywords:** optical interconnects, optical modulation, silicon modulators, fabrication variation

## Abstract

Driving signal reflection on traveling wave electrodes (TWEs) is a critical issue in Mach–Zehnder modulators. Fabrication variation often causes a random variation in the electrode impedance and the signal reflection, which induces modulation characteristics variation. The variation of reflection could be intertwined with the variation of other electrode characteristics, such as microwave signal attenuation, resulting in complexity. Here, we characterize the (partial) correlation coefficients between the reflection and modulation characteristics at different bit rates. Decreasing correlation at higher bit rates is observed. Device physics analysis shows how the observed variation can be related to nanoscale variation of material properties, particularly in the embedded diode responsible for electro-optic modulation. We develop a detailed theory to analyze two variation modes of the diode (P-i-N diode or overlapping P/N regions), which reveal insight beyond simplistic diode models. Microwave signal attenuation tends to reduce the correlation with on-electrode reflection, particularly at high bit rates. The theory shows the relative importance of conductor-induced attenuation and “dielectric”-induced attenuation, with different dependence on the frequency and fabrication variation. Strategies on how to mitigate the effect of variation for better fabrication tolerance are discussed by considering three key factors: pre-shift in structural design, bias condition, and fabrication control accuracy.

## 1. Introduction

Silicon-based optoelectronic devices, which are compatible with complementary metal oxide semiconductor processes, are becoming increasingly attractive [1,2], because they have the potential to provide high-bandwidth communications with low cost and low power consumption [3]. Silicon optical modulators are key components of silicon photonics [4,5]. Mach–Zehnder modulators (MZMs) with traveling wave electrodes (TWEs) have received significant attention, owing to their broad bandwidth and reliable operation [6,7,8,9,10,11,12,13,14,15,16]. In reality, fabrication processes often introduce substantial structure variations that are difficult to control. Indeed, fabrication variation is an important issue that needs to be investigated for the practical use of silicon photonics. A number of studies have considered the effect of the fabrication variation. Fabrication variation of passive devices has been extensively studied [17,18,19,20]. Due to its complexity, the variation of TWE modulators performance has seldom been analyzed in depth.

For TWE MZMs, the impedances of the electrodes and their terminations often vary in fabrication processes, resulting in a significant variation of optical modulation characteristics. In our previous work [21], we analyzed the correlation between the reflection of the driving signal and the characteristics of the output signal at a single bit rate. However, other key characteristics of TWEs, such as the attenuation of driving signal, could be intertwined with the impedance variation, causing difficulties in differentiating their individual effects. To fully understand such complex situations, further experiments and judicious quantitative analysis of low-level physical variation scenarios are needed. Note that the performance variation of modulators due to fabrication variation is crucial to the device yield, and hence it directly affects the actual device cost. As silicon modulators are key active devices in many applications, this problem can be an important factor in achieving the real promise of low cost for silicon photonics.

Here, we design a modulator with longer electrodes and investigate the correlation trend with increasing bit rates. The bit-rate dependence provides a new dimension to analyze the correlation behavior. The (partial) correlation coefficients between the driving signal reflection (*S*_11_) and modulation characteristic parameters, such as the bit error rate (BER), signal-to-noise ratio (SNR), extinction ratio (ER), and jitter, are characterized. The results show that with increasing modulation bit rate, the (partial) correlation coefficients decrease gradually. To account for the observed behavior, we develop a dedicated theory to analyze the variation of key device structures, particularly the TWE and the embedded diode with nanoscale features. Fabrication variation can produce unintended variation of material properties (e.g., spatial variation of dopant concentrations) at the scale of 25~100 nm, which can substantially affect the total impedance of the electrode and cause reflection variation. The implications of the results in improving the design and fabrication of silicon MZMs are discussed.

## 2. Structures and Methods

**Figure 1:**
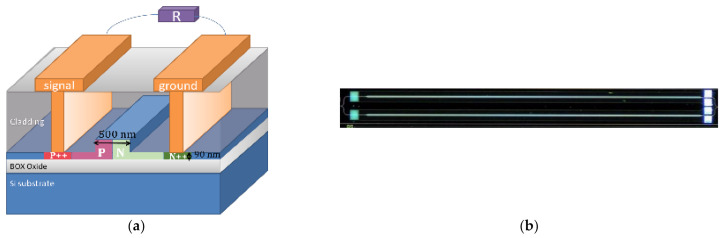
(**a**) Schematic of essential device structure and (**b**) optical microscopic image of a fabricated device.

Figure 1a illustrates a schematic of the modulator. Our devices were fabricated on a silicon-on-insulator wafer in a foundry with a top silicon layer thickness of 0.22 μm and a buried oxide thickness of 2 μm. The silicon ridge waveguide is 0.5 μm in width and 220 nm in height, with a 0.09 μm thick slab. The modulator was based on an asymmetric MZI structure and consisted of two phase shifters with different lengths of 20 μm between the arms, resulting in a free spectral range of ~30 nm. The light was coupled into and out of the chip by two grating couplers. Two multimode interference couplers (MMIs) were used as input/output 3 dB couplers. The modulation of MZMs was based on the carrier depletion.

A PN junction was designed with the junction interface at the center of the waveguide, and the P and N doping regions were doped to *N_A_* = 3.5 × 10^17^ cm^−3^ and *N_D_* = 2.3 × 10^17^ cm^−3^. The P and N slab regions both had a width of 1 μm. As we shall see, nanoscale variation of material doping concentration near the junction interface due to fabrication variation could play a key role in the device performance variation and will be one point of focuse point in this work. The P++- and N++-doped regions were 1 µm away from the edge of the ridge for ohmic contact. The position of these doped regions was designed to avoid guided mode propagation loss. The coplanar waveguide electrode was used with a pattern of ground signal ground signal (GSGS). The characteristic impedance of our device was designed to be around 50 Ohm, assisted by the coplanar waveguide design principle and finite element simulation [9,21,22]. The G and S electrodes made out of aluminum both had a width of 60 µm and a thickness of 0.75 µm, and the gap between them was 5.5 µm. A matched resistor was integrated onto the chip to reduce the reflection of the RF signal at the output.

Figure 1b shows an optical microscope image of the device and the length of the phase shifter is 4 mm. As we shall see, such a relatively long electrode makes it easier to observe a change in the reflection-induced effect at different bit rates.

**Figure 2:**
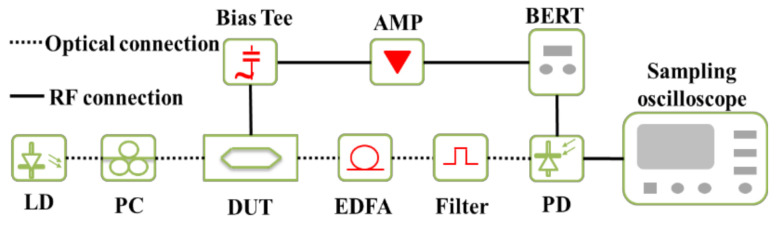
Experimental setup of measurement system used to characterize a Mach–Zehnder modulator (MZM).

The devices were characterized experimentally. The RF driving signal reflection was characterized by a Keysight vector network analyzer (VNA) with a proper calibration process to remove the contributions of the cable and the probe. The modulation experiment was performed with the setup shown in Figure 2. A bit error rate tester (BERT) was used to provide different bit rates of the pseudo random binary sequence (PRBS) signal with a pattern length of 2^31^–1. The driving voltage swing was 3.2 V and the dc bias voltage is −1.7 V. The signal was applied on the MZM through a 40 GHz GS probe. The light wave from a tunable laser was controlled by a polarization controller that then coupled into the device. The typical insertion loss of the modulator itself was ~5 dB, which includes the losses in MMIs and waveguide propagation loss in the phase shifters. The modulated optical signal, amplified by an erbium-doped fiber amplifier (EDFA), was fed into a commercial 50 GHz photodetector. The signal was then sent back to the BERT to test the BER or was captured by an Agilent sampling oscilloscope to measure the performance parameter. All devices were set to operate at the quadrature point and the power of the receiving signal was controlled (via setting EDFA power) to be fixed at ~9 dBm before entering the photodetector.

## 3. Results

**Figure 3:**
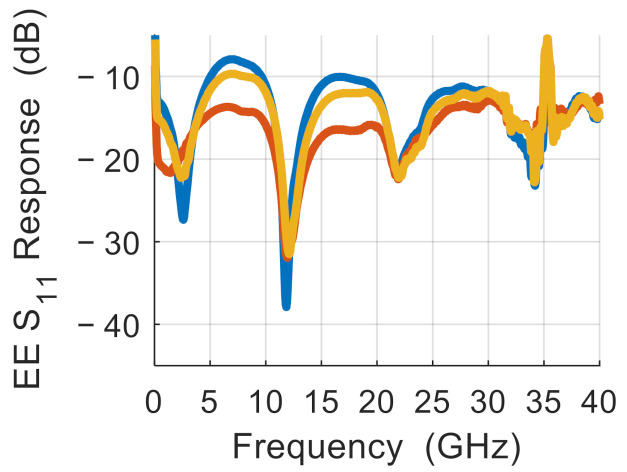
Representative *S*_11_ response (best: red; intermediate: yellow; worst: blue).

First, we characterize the driving signal reflection on the traveling wave electrode and the modulated signal output. The *S*_11_ response versus the frequency of the MZMs is measured under the 1.7 V reverse bias voltages (same voltage as in high-speed modulation experiments shown later). The representative *S*_11_ results are shown in Figure 3. The test data are measured from a number of devices (same design) fabricated in one batch. The *S*_11_ results indicate that all devices have varying degrees of mismatch between the traveling wave electrode and the terminal resistor due to fabrication variation.

**Figure 4:**
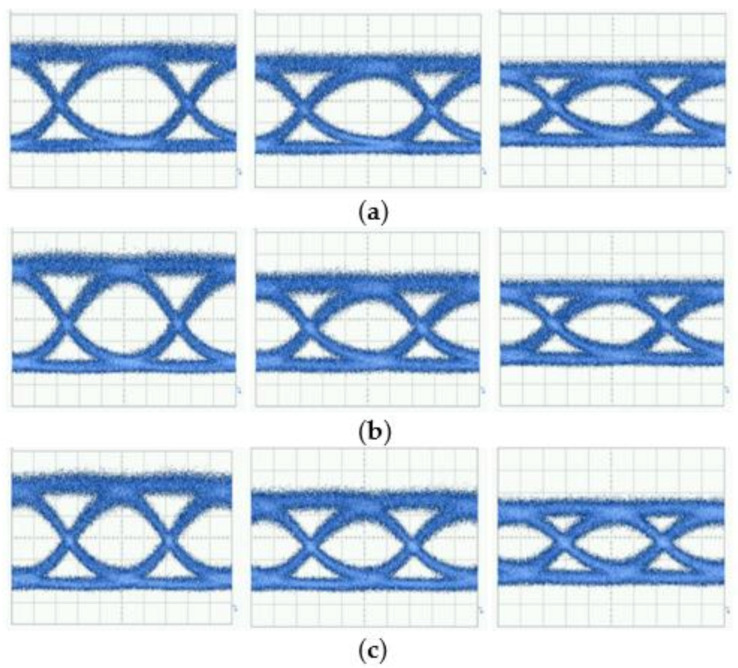
Representative eye diagrams at (**a**) 22, (**b**) 25, and (**c**) 28 Gb/s (from left to right: best, intermediate, worst). Unit for the horizontal axis: 8 ps/div; unit for the vertical axis: 20 mV/div.

Subsequently, we characterize the modulation performance of the devices at different bit rates. Figure 4 presents the results for the (a) 22, (b) 25 and (c) 28 Gb/s eye diagrams of the representative devices. It is clear that the modulation characteristics vary substantially.

**Figure 5:**
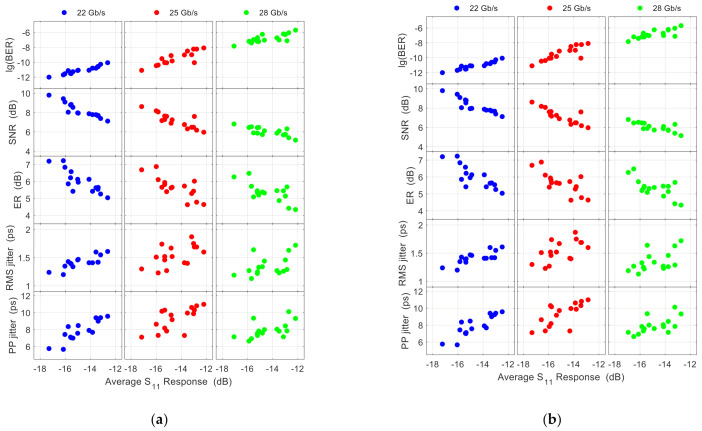
Relations between the bit error rate (BER), signal-to-noise ratio (SNR), extinction ratio (ER), root-mean-square (RMS) jitter, peak-to-peak (PP) jitter and the average of the *S*_11_ response over the frequency range of (**a**) 0.75 times the data rate and (**b**) equal to the data rate.

The relationship between the average *S*_11_ response (<*S*_11_>) and the performance parameters in two representative frequency ranges is shown in Figure 5a,b. The frequency range for averaging *S*_11_ in Figure 5a is roughly 0.75 times the data rate, which contains most of the signal power [23]. The frequency range for averaging *S*_11_ in Figure 5b is equal to the data rate. In both figures, the relation of BER vs. <*S*_11_> is almost a straight line at 22 Gb/s. As the data rate increases, some deviation from the straight line becomes increasingly obvious. Similar behaviors happen to the SNR vs. <*S*_11_>, although at 22 Gb/s, the deviation is already quite visible. Generally, the relationships between other parameters of the modulated signal and <*S*_11_> have similar trends as the data rate increase, although the degrees of irregularity of data points may vary in each case. For example, it can be difficult to ascertain a clear trend between *S*_11_ and root-mean-square (RMS) jitter and peak-to-peak (PP) jitter at 28 Gb/s. Comparing Figure 5a,b, the trend of changing deviation with the data rate (from left to right) is similar. Generally, when the averaging frequency range contains most of the signal power, this trend does not change. Hence, we will use a fixed frequency range in subsequent analysis.

To quantify the relationship between <*S*_11_> and performance parameters, we calculate the Pearson correlation coefficient *ρ* (<*S*_11_>, x), where x = BER, SNR, ER, RMS jitter or PP jitter:

(1) $$\rho \left(y,x\right)=\frac{\sum_{i}\left(y_{i}-\bar{y}\right)\left(x_{i}-\bar{x}\right)}{\sqrt{\sum_{i}{\left(y_{i}-\bar{y}\right)}^{2}\sum_{i}{\left(x_{i}-\bar{x}\right)}^{2}}}$$

where *i* is the index of the devices. Note that the amplitude of the modulated signal ∆*P* (the difference between states “1” and “0”) varies with the device.

To remove the influence of the amplitude variation, we can calculate the partial correlation coefficient [20]. The partial correlation coefficient between two parameters, *y* and *x*, while holding the third parameter *z* (=∆*P* here) constant was calculated via the standard form [24]:

(2) $$p\left(y,x;z\right)\equiv \frac{\left[\rho \left(y,x\right)-\rho \left(y,z\right)\rho \left(x,z\right)\right]}{{\left\{\left[1-\rho \left(y,z\right)\right]\left[1-\rho \left(x,z\right)\right]\right\}}^{\frac{1}{2}}}$$

The correlation coefficients *ρ* (<*S*_11_>, ∆*P*), *ρ* (*x*, ∆*P*) in the formula of *p* (<*S*_11_>, *x*; ∆*P*) can be readily calculated.

**Figure 6:**
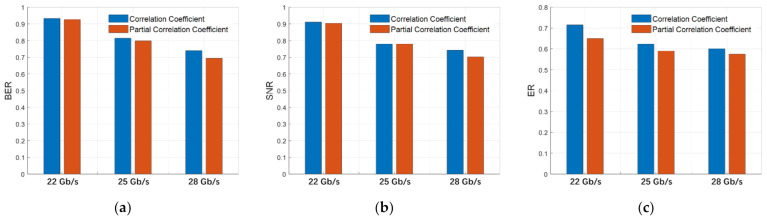
Absolute value of the correlation coefficients and partial correlation coefficients of average *S*_11_ with performance parameters: (**a**) BER, (**b**) SNR, and (**c**) ER.

To illustrate how the correlation changes with data rate, we plot the (partial) correlation coefficients between <*S*_11_> and the strongly correlated performance parameters (BER, SNR, and ER) in Figure 6. Here, *S*_11_ is averaged over the full frequency range (*f_max_* = 40 GHz) of our test system. Choosing another averaging frequency range (as long as it contains most of the signal power) will result in a similar trend. It is evident that both the correlation coefficient and the partial correlation coefficient decrease as the modulation rate increases. Generally, Figure 5 and Figure 6 show that BER, SNR, and ER are more affected by the RF signal reflection, whereas the signal timing (jitters) is less affected, which is understandable as the driving signal reflection directly affects the signal amplitude and has only a secondary influence on the signal timing through non-steep transition edges [21]. Note that the reflected signal is superposed on the original driving signal. As the reflected signal often carries the information from the previous bit(s), it may randomly increase or decrease the amplitude of the current bit of the driving signal. Hence the reflection generates noise, which explains its correlation with SNR. The BER is often directly related to the SNR (via well-known formulas, see Ref. [25] for example). The ER variation originates largely from the nonlinear relation between driving voltage and modulated output signal [21].

To understand the decreasing correlation with increasing data rate, we note that the reflected signal on the electrode is superposed on the original driving signal, which can distort the original signal, thus causing different effects on the various characteristics of the modulated signal in these devices. With increasing modulation data rate, the fraction of high-frequency content in the driving signal will increase. When the signal reaches the TWE end and is reflected, the reflected signal tends to be weaker because more high-frequency content experiences more attenuation, and the reflected signal will be attenuated more along the reverse propagation path and thus produces fewer effects. Although the forward propagating driving signal is also attenuated, its attenuation is less than the reflection attenuation. For example, if the attenuation of the forward signal is 0 to *u* dB from the beginning to the end of the TWE, the reflected signal will be attenuated by *u* to 2*u* dB from the end of TWE to the beginning. Obviously, the reflected signal is attenuated more. To further illustrate this, one can imagine an extreme case that the high frequency signal is attenuated to almost zero at the TWE end, the reflection would have virtually no effect on the device performance. In addition, the reflected RF signal is counter-propagating on the electrode. As such, theory shows that the modulation effect due to reflected RF signal typically decreases with frequency (with a sinc function type of behavior in some simplified models) [26], which suggests a reduced modulation bandwidth of the reflected signal compared to that of the original driving signal. This is another reason that the effect of reflection is less for faster bit rates. Therefore, the relative contribution of reflection might become less at high frequencies (or high data rates), which suggests lower correlation.

## 4. Discussion

To fully understand where the variation of reflection comes from, we need delve into the underlying structure and investigate how the variation of TWE structure parameters influences the reflection. The output power for MZMs depends on the electro-optic phase shift Δφ as $P=P_{0}+P_{1}\cos\;\left(\Delta \varphi -\Delta \varphi_{0}\right)$, where *P*_0_, *P*_1_ and $\Delta \varphi_{0}$ are constants. The phase shift Δφ is proportional to the refractive index change Δn, which depends on the effective voltage on the TWEs. The effective voltage on the TWEs is affected by the reflected signal caused by the terminal mismatch, resulting in the difference of the modulation performance parameters between the devices, as shown in Figure 4. As shown in Figure 2, the peak value of the *S*_11_ difference between the best and worst devices is over 5 dB. Note that the terminal mismatch is caused by the variation in electrode impedance and terminal resistance. Based on the DC measurement, the resistance of the terminating resistor varies between 48 and 53 Ω. The electrode impedance is related to a large number of large structural features of the electrode (including the gap and thickness) and small features within the optical waveguide (such as the diode with nanoscale junction width), as shown in Figure 1a. Note that the variation of structural dimensions is typically at the scale of 25~100 nm for some key features. In addition, the degree of influence that each parameter exerts on its respective structure may differ. However, with the presence of the on-chip termination, it is difficult to directly measure the frequency-dependent total impedance of each individual device.

In order to find how the variation of the impedance is related to the variation of the low-level structure parameters, a widely used modeling method [8,14,26,27] is employed here. In this method, the PN loaded characteristic impedance *Z*_0_ and propagation constant γ can be expressed as:

(3) $$Z_{0}=\sqrt{\frac{R_{tl}+j\omega L_{tl}}{\left(G_{tl}+R_{tpn}^{-1}\right)+j\omega \left(C_{tl}+C_{tpn}\right)}}.$$

(4) $$\gamma =\alpha +j\beta =\sqrt{\left(R_{tl}+j\omega L_{tl}\right)\left(\left[G_{tl}+R_{tpn}^{-1}\right]+j\omega \left[C_{tl}+C_{tpn}\right]\right)}$$

where *R_tl_*, *L_tl_*, *C_tl_* and *G_tl_* are the resistance, inductance, capacitance, and leakage conductance of the transmission line per unit length, respectively, *α* is the amplitude attenuation constant, *β* is the phase constant, and *R_tpn_* and *C_tpn_* are the parallel equivalent values of the diode’s resistance and capacitance, respectively. Finite-element method (FEM) simulations in the 2D cross-section of the modulator are performed to obtain the transmission line parameters of the electrodes. The P-N diode’s characteristics are largely governed by a one-dimensional differential equation along the horizontal axis, which can be readily solved with well-established analytic formula [28].

**Figure 7:**
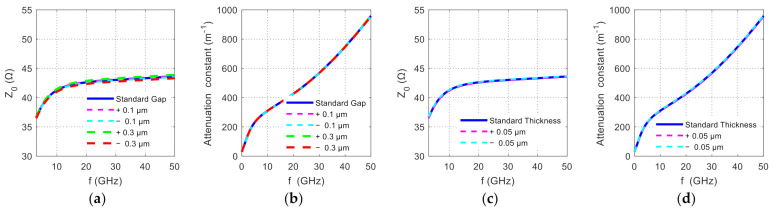
Simulated variation of the TWE with the P-N junction loaded: (**a**) total impedance and (**b**) total power attenuation constant varying with the gap between G and S; (**c**) total impedance and (**d**) total power attenuation constant varying with the electrode thickness.

Considering that the width variation of the electrode metal dimensions is about 2%, we simulated the influence of the change in the gap between the metal electrode. In Figure 7, the blue solid line is the simulation result obtained at the initial design value. Figure 7a,b shows the change in the total impedance of the PN junction loaded transmission line and the attenuation constant when the electrode gap is changed. When the frequency is lower than 10 GHz, there is almost no difference in the impedance and the attenuation constant of different gap variations. In the full test frequency range, when the gap change is ±0.3 μm, the total impedance changes by about ±0.3 Ω. Note that for such a gap (5.5 μm), the range of width variation (maximum minus minimum) is generally substantially less than 10% [29]. The variation in the gap of the electrode caused by the fabrication variation has minimal effect on the variation of the transmission loss of the TWEs. Note that because the electrode width is one order of magnitude larger than the gap width, the electrode width variation (while the gap width is fixed) produces a negligible effect compared to the gap width variation. In addition, we also simulate the influence of the change in thickness of the TWEs. Typically, the range of metal film thickness variation should be less than 10% [29]. Figure 7c,d show the simulation results for the thickness varying by ±0.05 μm. As the thickness of the electrode varies by 0.05 μm, the impedance of the PN junction loaded transmission line changes is around 0.1 Ω in the test frequency range. The variation of the microwave attenuation influenced by the variation of the thickness is around −1%. In general, the variation of the electrode’s structural characteristics has a very small effect on the transmission line impedance (±1%), which cannot account for the observed over 5 dB of variation of RF signal reflection.

**Figure 8:**
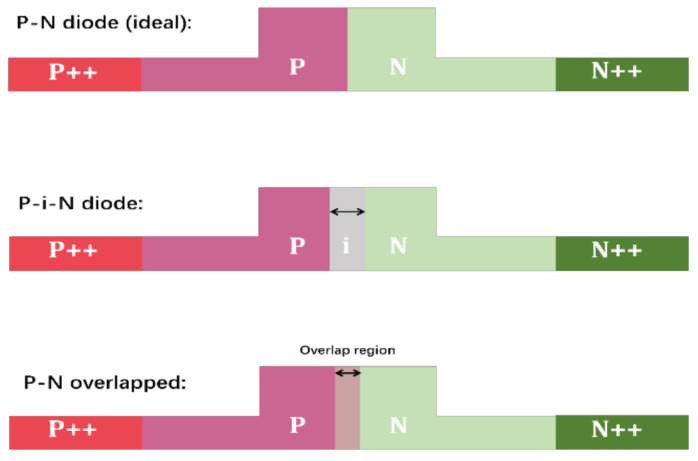
Two fabrication variation scenarios of the diode.

Compared to the features of the electrodes, the edges of the P and N regions in submicron optical waveguides are more likely to shift due to multiple processing steps. For example, the edge of each region may obviously shift due to misalignment during the lithography. Furthermore, the width of the P or N region may expand or shrink during the lithography and etching steps, which may also cause the edge of each region to shift. The edge shift of the P and N doping regions causes unintended spatial variation of materials property (i.e., dopant concentration). Although the linewidth variation and misalignment can be controlled to the order of 25~100 nm, this may still have a relatively large effect on the capacitance of the PN junction because the junction width itself is of the order of 100 nm. As shown in Figure 8, fabrication variation may cause two variation scenarios of the diode. The first type is the diode becoming a P-i-N diode (i.e., with a nano-gap between the P and N regions), and the second type causes the diode to have a nanoscale overlapping region. Either case can cause substantial junction capacitance variation.

**Figure 9:**
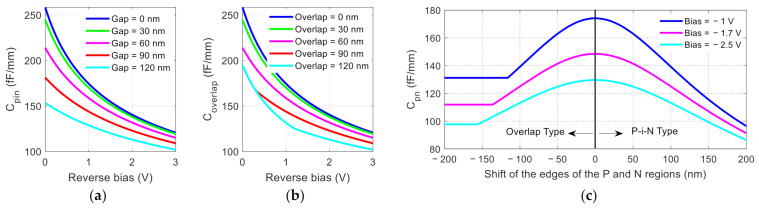
Calculated variation of junction capacitance with bias voltage for (**a**) P-i-N type and (**b**) overlap type; and (**c**) diode under different biases for both types.

For the first type, the junction capacitance can be modeled by a relatively simple formula [28]:

(5) $$C_{pin}=C_{pn}\frac{1}{\sqrt{1+W_{i}^{2}/W_{pn}^{2}}}$$

where *W_i_* is the nano-gap width between the P and N regions and *Wpn* and *Cpn* are the junction width and capacitance of a corresponding PN diode, respectively. Figure 9a shows the junction capacitance variation with bias voltage for different gap widths. At the same bias voltage, different gap widths give rise to different junction capacitance drop rates (e.g., when the reverse bias voltage is 1 V, the junction capacitance drops by ~5 fF/mm when the gap is increased from 0 to 30 nm and by ~15 fF/mm when the gap is increased from 90 to 120 nm). In addition, when the bias voltage is smaller, the junction capacitance changes at a faster rate with the change in gap width.

For the second type, the overlapping region will be P doped with a net concentration of $N_{A^{-}}=N_{A}-N_{D}=1.2\times 10^{17}cm^{-3}$. Here, two cases must be considered. First, if the overlap region is wide and the bias voltage is small, the overlap region may not be fully depleted. In this case, the capacitance can be readily calculated for a PN junction where the P side is doped to a concentration of *N_A_^−^*. Second, if the overlap region is narrow and the bias voltage is sufficient to deplete the overlap region to reach the fully doped P region, the capacitance of such a junction is more complicated. For this type, detailed derivation in the Appendix A shows that the junction capacitance is:

(6) $$C_{ov}=\frac{\varepsilon_{Si}}{\sqrt{\gamma_{A}^{2}w_{ov}^{2}+\frac{2\varepsilon_{Si}}{qN_{D}}\gamma_{A}\left(\psi_{0}-V\right)}+\sqrt{\gamma_{D}^{2}w_{ov}^{2}+\frac{2\varepsilon_{Si}}{qN_{A}}\gamma_{D}\left(\psi_{0}-V\right)}}$$

where *q* is the electron charge, *ε_Si_* is the dielectric constant of silicon, *w_ov_* is the overlap width, *ψ_0_* is the built-in potential, *V* is the bias. The definition of *γ_A_* and *γ_D_* is given in Equation (A8) in Appendix A. Figure 9b shows the variation of junction capacitance with bias voltage for different overlap widths. Different from the P-i-N diode scenario, when the overlap area is large, the curves may merge (for example, when the reverse bias was less than 0.4 V, the junction capacitance curves corresponding to an overlap of 90 nm and an overlap of 120 nm have the same value). It can be shown that this happens due to the first case where the small bias is not sufficient to deplete a 90 nm wide overlap region. Hence, when the overlap width is increased to 120 nm, the depletion region is not affected, and the capacitance remains the same.

Figure 9c shows the junction capacitance changes in the two scenarios of variations under different bias voltages (positive or negative shift of the edges of the P and N regions corresponds to the P-i-N or overlap scenario, respectively). Both variation scenarios of the diode lead to a reduction in PN junction capacitance. Furthermore, for the P-i-N case, the capacitance continues to decrease as the i region increases, while for the overlap scenario, when the overlap region is very large, the capacitance reaches a stable value (indicating that the voltage is insufficient to deplete the overlap region).

**Figure 10:**
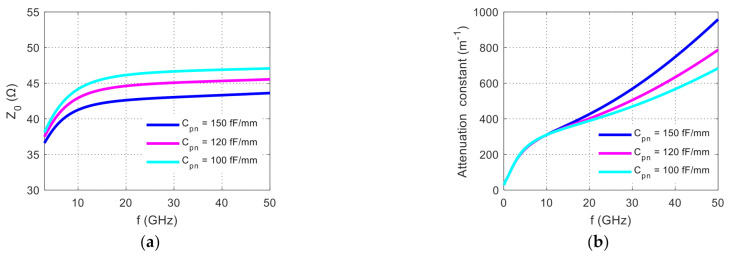
Simulation results of (**a**) total impedance and (**b**) total power attenuation constant varying with the junction capacitance.

Figure 10a shows the FEM simulation result of the total impedance changes corresponding to the *C_pn_* changes. If the individual shift of the P and N edges is ~100 nm (hence the worst relative shift under opposite shift directions is ~200 nm), the total impedance of the electrode increased by 8% or more, which can produce substantial reflection variation as observed experimentally. Compared with the change in the electrode parameters, the change in the impedance caused by the change in the location of the P and N regions is more significant, which is a major factor for the reflection variation between the devices.

In order to better understand variation of RF attenuation due to the fabrication variation of the diode, we note that the amplitude attenuation constant can be roughly approximated as [14]:

(7) $$\alpha =\frac{1}{2}\left(R_{DC}+R_{AC}\sqrt{f}\right)\sqrt{\frac{C_{tl}+C_{pn}}{L_{tl}}}+\frac{1}{2}\left(4\pi^{2}f^{2}C_{pn}^{2}R_{pn}\right)\sqrt{\frac{L_{tl}}{C_{tl}+C_{pn}}}$$

where the first term is the loaded conductor loss, the second term is the silicon “dielectric” loss, and *R_DC_* and *R_AC_* are some effective constants. Note that the “dielectric” loss increases substantially with the PN junction capacitance and with frequency according to Equation (7). When the junction capacitance changes, the transmission loss also changes. Here, we calculate the total attenuation constant and plot it in Figure 10b. When the frequency is low (<15 GHz), the difference in the total microwave attenuation constant caused by the change in the *C_pn_* is negligible. As the frequency increases, the variation in the total microwave attenuation constant gradually increases. This can be clearly understood through Equation (7). At low frequencies, the conductor loss (~$\sqrt{f}$) given by the first term dominates, whereas at high frequencies, the “dielectric” loss (~*f*^2^) given by the second term dominates. One readily shows that the *C_pn_*^2^ factor in the second term gives much higher relative variation than the $\sqrt{C_{tl}+C_{pn}}$ factor. Hence the overall loss exhibits more variation at high frequencies where the “dielectric” loss dominates. As most signal power is contained in the relatively low frequency range, the power in the frequency range above 15 GHz is less than ~15% for 22~28 Gb/s signals. Hence, the effective attenuation variation is fairly small (estimated < ~1.5%) for the modulated output signals. Note that impedance variation ~10% shows up at a much lower frequency (~7 GHz) and continues to high frequencies, affecting around half of the signal power. Hence, the effective impedance variation is fairly substantial.

**Figure 11:**
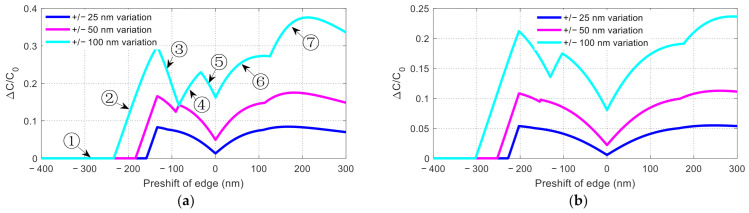
Mitigation of fabrication variation: Junction capacitance affected by the edge shift of the P and N regions caused by fabrication variation for different relative pre-shift of the edges. (**a**) under −1.7 V bias, (**b**) under −5 V bias. *C*_0_ = *C_pn_* is the original PN junction capacitance with no overlap or gap. Positive pre-shift: P-i-N case. Negative pre-shift: overlap case.

The reflection variation of the driving signal on the TWEs is one of the most common fabrication variations in silicon MZMs. In order to mitigate the modulation characteristics variation due to the on-electrode reflection variation, more emphasis should be placed on reducing diode variation. According to the above analysis, the variation of the capacitance of the PN junction is affected by the fabrication control accuracy and the bias voltage, hence proper setting of these two parameters may help mitigate the effect of reflection variation. Furthermore, it is possible to pre-shift edges of the P and N regions in design towards the goal of avoiding some worst scenarios of the relative shift. To find promising strategies, Figure 11 shows the maximum variation of the junction capacitance when the P and N region’s edges with different pre-shifts are subjected to different fabrication control accuracy (i.e., different fabrication variation). Two representative bias voltages are considered in Figure 11a,b. Overall, the left half of each curve is generally lower than the right half (divided at the zero pre-shift), which suggests that negative pre-shift or the overlap case is preferred to reduce junction capacitance variation. A typical curve can be divided into seven segments by cusp points. At each cusp point, the worst variation case swaps from the negative fabrication-induced shift to positive, or vice versa.

To mitigate the capacitance variation at a bias voltage −1.7 V, for a maximum fabrication variation of ±100 nm, a pre-shift of −80 nm between the P and N regions appears to be a fairly good setting, indicated by a local minimum on the top curve in Figure 11a. This means that a small overlap region (80 nm wide) can be designed in advance to control the manufacturing deviation. For better nanofabrication control (i.e., smaller fabrication variation) of ±25 and ±50 nm, zero pre-shift (i.e., no intentional shift) appears to be a better choice according to the local minimum of the respective curves, which is beyond our expectations (preliminary exploration focused on the P-i-N case only [21] is inclined to better tolerance with a sufficiently wide i region). Note that the ±100 nm curve almost touches the ±50 nm curve at a pre-shift of −80 nm. This is an interesting exception that occurs accidentally due to: (1) a constant capacitance for the relative shift < −130 nm (=−80–50 nm) and (2) the peak at zero shift (within 100 nm from the pre-shift of 80 nm), as can be inferred from Figure 9c. To mitigate the capacitance variation to nearly zero, there is always the option of creating a large overlap region for the PN diode, as shown by the left-most segment (Segment 1) in Figure 11a. As shown in Figure 11a, the capacitance remains constant in the left-most segment. In this scenario, the carriers in the large overlap region will not be depleted at a given reverse bias and “break through” to the P doped region will not occur even with fabrication variation. As a result, the junction capacitance has the best fabrication tolerance. However, the large overlap approach may have some implications, which will be more obvious in subsequent discussion on high-bias scenarios.

As some applications may have a modulator working at a high bias voltage (e.g., [30]), we also show the capacitance variation under a larger bias voltage (−5 V) in Figure 11b. It can be seen that when the bias voltage is larger, the capacitance generally varies less for the same fabrication variation compared to Figure 11a. Intuitively, a larger bias tends to create a wider junction depletion region, which is less sensitive to P/N edge shift. Mathematically, this can be attributed to the fact that the capacitance generally contains $\sqrt{c_{0}\left(V_{0}-V\right)}$ type of dependence on voltage (in denominator), both in the P-i-N and overlap case, as shown in Equations (5) and (6). Note that *C_pn_* in Equation (5) contains a factor ~1/$\sqrt{c_{0}\left(V_{0}-V\right)}$. One can readily show that such a kind of voltage dependence tends to render the capacitance insensitive at high bias. Note that at larger bias voltages, if one chooses to create a wide overlap region to achieve near zero capacitance variation, the required overlap width may be too large (e.g., 300 nm of edge pre-shift is required at a relative edge shift change of ±100 nm at −5 V). As such, the P and N region edges may nearly touch the sidewall of the waveguide (only 100 nm margin on each side for a 500 nm wide waveguide), which may cause difficulty for carrier transport into the upper portion of the waveguide ridge. To avoid this, one may need better fabrication control (e.g., ±50 or 25 nm, indicated by the two lower curves in Figure 11b, so that one can work at the local minimum at zero pre-shift.

In this work, we take a hybrid approach to the fabrication-induced variation of the Radio Frequency (RF) signal reflection on the modulator’s electrode. At the device level, we adopt a more experimental approach to find the correlation between variation of the modulation characteristics and that of the reflection. At the low level (structure level), we use theoretical/numeric analysis to explore the underlying variation inside the electrode (plus the diode) that can contribute to the reflection variation. Such an approach helps to overcome some difficulties at the device level and at the structure level. At the structure level, the low-level structure variation (especially, the diode junction variation) is often hard to measure experimentally and extensively. At the device level, modeling of the large-signal digital modulation for the silicon modulator is substantially more complicated, considering the nonlinear dependence between the driving signal and output modulated signal, pseudo-random bit sequence and other complications. Building a full simulation software package from low-level structure variation to digital modulation characteristics (e.g., BER, SNR) variation is beyond the scope of this work. Note that the widely used modeling method of the electrode impedance for junction-loaded modulators [8,14,26,27] is also a hybrid method that combines a direct solution of the Maxwell equation for the metal electrode plus an equivalent circuit model for the loaded diode. A hybrid approach divides a complicated problem into several parts so that each part can be solved by the most effective method, which is often useful in tackling some very complicated problems.

## 5. Conclusions

This work investigates the correlation between driving signal reflection variation on the electrode and modulation characteristics variation at different bit rates. Decreasing correlation with increasing bit rates is observed and is attributed to increasing high-frequency content at higher bit rates, for which reflection produces fewer effects due to increased signal attenuation and due to the smaller bandwidth of counter propagating interaction.

Low-level theoretical/numeric analysis of the underlying structures shows that the reflection variation on the electrode is less affected by the fabrication variation of the electrode itself and more affected by the variation in the embedded diode, particularly nanoscale variation of material doping concentrations. We develop a detailed theory to analyze the two modes (P-i-N or overlap) of variation of the embedded diodes. It is shown that the capacitance variation is influenced by the fabrication control accuracy and the bias voltage.

We also provide strategies on how to reduce the effects of fabrication variation analyzed herein. When the reverse bias voltage is small, a diode with a relatively narrow overlapping P/N region (e.g., 80 nm at −1.7 V) might be a reasonably good design choice when the fabrication variation is large. With reduced fabrication variation (e.g., 25~50 nm), an “aboriginal” diode design with zero pre-shift of P/N edges appears to be reasonably good. Generally, a sufficiently large overlap region could in principle achieve zero capacitance variation, although the required overlap width may be excessively large under large bias voltages, leading to other concerns.

The methodology and results of this work may also help in studying the fabrication variation of other integrated photonic devices.

## Data Availability

The data presented in this study are available on request from the corresponding author W.J.

## Appendix A

To study the overlap scenario of a diode, we consider the following model. For the P region and P^−^ region side, the acceptor concentration is *N_A_*(*x*) is a step function comprising two segments of constant values ($N_{A}$ and $N_{A^{-}}=N_{A}-N_{D}$). If the voltage is small, such that the overlap region is not fully depleted, this junction can be easily treated as a PN junction. However, if the overlap region is fully depleted and the depletion region extends to the P region, the simple PN junction capacitance formula no longer applies. In this case, the relative potential for the PP^−^ side becomes:

(A1) $$\psi_{P}=\frac{-q}{\varepsilon_{Si}}\int_{-x_{p}}^{0}xN_{A}(x)dx$$

(A2) $$\psi_{P}=\left(q/\varepsilon_{Si}\right)\left[N_{A}x_{p}^{2}/2-N_{D}x_{ov}^{2}/2\right]$$

where *q* is the electron charge, *ε_Si_* is the dielectric constant of silicon, −*x_p_* is the location of the depletion edge in the P region, *w_ov_* is the overlap width. The relative potential of the N side is:

(A3) $$\psi_{N}=\frac{q}{\varepsilon_{Si}}\int_{0}^{x_{n}}xN_{D}\left(x\right)dx=\frac{q}{\varepsilon_{Si}}N_{D}\left(x_{n}^{2}/2\right)$$

where *x_n_* is the location of depletion edge in the N region. The total potential across the junction satisfies the relationship:

(A4) $$\psi_{P}+\psi_{N}=\psi_{0}-V$$

where *V* is the bias and the built-in potential is given by:

(A5) $$\psi_{0}=\frac{1}{2}\frac{kT}{q}In\left(\frac{N_{A}^{2}N_{D}^{2}}{n_{i}^{4}}\right)$$

where *k* is the Boltzmann constant, *T* the temperature, and *n_i_* is the intrinsic carrier concentration. In thermal equilibrium, the total negative charge per unit area in the P side and P^−^ side must be equal to the total positive charge per unit area in the N side:

(A6) $$N_{A}\left(x_{p}-w_{ov}\right)+\left(N_{A}-N_{D}\right)w_{ov}=N_{D}x_{n}$$

Combining (11), (12), (13), we have

(A7) $$N_{D}{\left(x_{n}+w_{ov}\right)}^{2}+N_{A}\left(x_{n}^{2}-w_{ov}^{2}\right)=\frac{N_{A}\left(2\varepsilon_{Si}/q\right)\left(\psi_{0}-V\right)}{N_{D}}$$

which gives *x_n_* as an implicit function of *V*. In addition, according to (13), $x_{p}=\left(N_{D}/N_{A}\right)\left(x_{n}+w_{ov}\right)$, and note that the depletion width is given by $W=x_{p}+x_{n}$. Through lengthy calculation, one can obtain the depletion width *W*(*V*) as a function of voltage

(A8) $$W\left(V\right)=\sqrt{\gamma_{A}^{2}w_{ov}^{2}+\frac{2\varepsilon_{Si}}{qN_{D}}\gamma_{A}\left(\psi_{0}-V\right)}+\sqrt{\gamma_{D}^{2}w_{ov}^{2}+\frac{2\varepsilon_{Si}}{qN_{A}}\gamma_{D}\left(\psi_{0}-V\right)}$$

where

(A9) $$\gamma_{A}=\frac{N_{A}}{N_{A}+N_{D}},\;\gamma_{D}=\frac{N_{D}}{N_{A}+N_{D}}$$

The result of the depletion layer capacitance is $C\left(V\right)=\varepsilon_{Si}/W\left(V\right)$.
